# Modelling the conditional regulatory activity of methylated and bivalent promoters

**DOI:** 10.1186/s13072-015-0013-9

**Published:** 2015-06-19

**Authors:** David M. Budden, Daniel G. Hurley, Edmund J. Crampin

**Affiliations:** Systems Biology Laboratory, Melbourne School of Engineering, The University of Melbourne, 3010 Parkville, Australia; NICTA Victoria Research Laboratory, The University of Melbourne, 3010 Parkville, Australia; ARC Centre of Excellence in Convergent Bio-Nano Science and Technology, 3010 Parkville, Australia; Department of Mathematics and Statistics, The University of Melbourne, 3010 Parkville, Australia; School of Medicine, The University of Melbourne, 3010 Parkville, Australia

## Abstract

**Background:**

Predictive modelling of gene expression is a powerful framework for the *in silico* exploration of transcriptional regulatory interactions through the integration of high-throughput -omics data. A major limitation of previous approaches is their inability to handle conditional interactions that emerge when genes are subject to different regulatory mechanisms. Although chromatin immunoprecipitation-based histone modification data are often used as proxies for chromatin accessibility, the association between these variables and expression often depends upon the presence of other epigenetic markers (e.g. DNA methylation or histone variants). These conditional interactions are poorly handled by previous predictive models and reduce the reliability of downstream biological inference.

**Results:**

We have previously demonstrated that integrating both transcription factor and histone modification data within a single predictive model is rendered ineffective by their statistical redundancy. In this study, we evaluate four proposed methods for quantifying gene-level DNA methylation levels and demonstrate that inclusion of these data in predictive modelling frameworks is also subject to this critical limitation in data integration. Based on the hypothesis that statistical redundancy in epigenetic data is caused by conditional regulatory interactions within a dynamic chromatin context, we construct a new gene expression model which is the first to improve prediction accuracy by unsupervised identification of latent regulatory classes. We show that DNA methylation and H2A.Z histone variant data can be interpreted in this way to identify and explore the signatures of silenced and bivalent promoters, substantially improving genome-wide predictions of mRNA transcript abundance and downstream biological inference across multiple cell lines.

**Conclusions:**

Previous models of gene expression have been applied successfully to several important problems in molecular biology, including the discovery of transcription factor roles, identification of regulatory elements responsible for differential expression patterns and comparative analysis of the transcriptome across distant species. Our analysis supports our hypothesis that statistical redundancy in epigenetic data is partially due to conditional relationships between these regulators and gene expression levels. This analysis provides insight into the heterogeneous roles of H3K4me3 and H3K27me3 in the presence of the H2A.Z histone variant (implicated in cancer progression) and how these signatures change during lineage commitment and carcinogenesis.

**Electronic supplementary material:**

The online version of this article (doi:10.1186/s13072-015-0013-9) contains supplementary material, which is available to authorized users.

## Background

Understanding the precise spatiotemporal regulation of eukaryotic gene expression is a central challenge in molecular biology. Three features are common to the epigenetic interactions that underlie transcriptional regulatory programs [1]: sequence-specific recruitment of regulatory factors to binding sites (e.g. transcription factors and non-coding RNAs); enhanced specificity of regulatory function through the cooperative interactions of several factors; and the establishment of positive and negative feedback mechanisms (corresponding with post-translational histone modifications and DNA methylation) to stabilise targeted activity and facilitate its propagation through cell division. Dysregulation of these epigenetic interactions has been implicated with hundreds of developmental, autoimmune, neurological, inflammatory and neoplastic disorders [2].

Characterising gene regulatory programs by studying individual protein–protein interactions would require a currently unavailable volume and resolution of proteomics data. Instead, predictive modelling frameworks [3–7] have been developed that leverage the wealth of high-throughput sequencing data generated by recent large-scale consortia (e.g. ENCODE [8]) to predict the (indirect) relationships between transcription factors, epigenetic modifications and RNA transcript abundance. The utility of these models is not in the ability to predict RNA transcript abundance at the level of individual genes, but in the biological insights into gene expression regulation that can be gained by exploring genome-wide relationships. Examples of downstream analysis include: inferring regulatory roles of transcription factors from their respective binding motifs [9]; identifying regulatory elements responsible for differential expression patterns [10]; exploring the relationship between gene expression and higher-order chromatin domains [11]; and large-scale comparative analysis of the transcriptome across distant species [12]. In each of these examples, the gene-level prediction accuracy is used as an indirect measure of the model’s explanatory potential.

Despite the utility of predictive modelling as a framework for exploring fundamental molecular biology, a major limitation of current approaches is their inability to model the conditional associations that emerge between histone modifications and gene expression in the presence of other epigenetic markers (e.g. in methylated or bivalent promoters). To highlight this shortcoming, we formulate and evaluate several methods of quantifying promoter-localised DNA methylation and demonstrate that its naïve integration into previous models is unable to improve prediction accuracy. These results are due to statistical redundancy between DNA methylation and histone modification data (previously studied in transcription factors [11]) despite substantial anti-correlation between gene expression and promoter methylation levels.

**Fig. 1:**
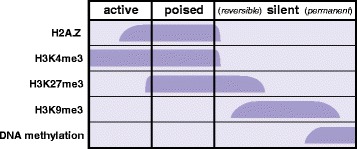
Illustration of the histone/epigenetic code in the context of the promoter-localised regulatory elements analysed in this study. Only active genes exhibit significant expression, corresponding with H3K4me3 often flanked by H2A.Z. Poised and reversible/permanently silenced genes are distinguished by decreasing likelihood of genes returning to an active state; poised genes are marked by bivalent H3K4/27me3 and H2A.Z, while silent genes are marked by H3K27me3 (facultative heterochromatin), H3K9me3 (constitutive heterochromatin) and DNA methylation (permanent silencing)

In this study, we introduce a modelling framework that allows the integration of conditional regulatory data by unsupervised identification of latent regulatory classes. We demonstrate that this approach is effective at identifying gene silencing events (promoter methylation) and isolating the heterogeneous roles of H3K4me3 and H3K27me3 conditioned upon the H2A.Z histone variant (promoter bivalency), leading to substantial improvements in genome-wide accuracy of gene expression predictions. Specifically, our model integrates high-throughput sequencing data for DNA methylation, H3K4me3, H3K27me3, H3K9me3 and H2A.Z to predict mRNA transcript abundance levels (RNA-seq) across all ENCODE Tier1 cell lines [8]. A simplified histone/epigenetic code for these modifications in a promoter-localised context is illustrated in Fig. 1. The remaining histone modifications available on ENCODE are unsuitable for this study as they either target non-promoter regions (e.g. H3K36me3 in the 3′-UTR [13]) or are mutually exclusive and thus highly redundant with those selected for this study (e.g. H3K9/27 ac).

## Results and discussion

### Standard predictive modelling is unable to derive the regulatory signature of the H2A.Z histone variant

**Fig. 2:**
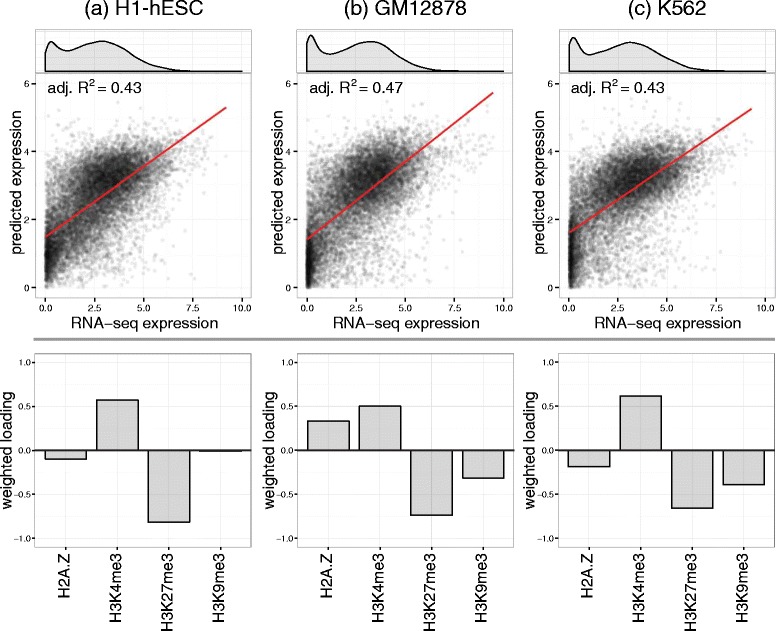
Analysis of predictive models of genome-wide transcript abundance for **a** H1-hESC, **b** GM12878 and **c** K562 cell lines, constructed from H2A.Z, H3K4me3, H3K27me3 and H3K9me3 histone scores. Each cell line demonstrates the following: (*top*) the distribution of arsinh-transformed RPKM-normalised transcript abundance derived from RNA-seq data; (*middle*) predicted-versus-measured transcript abundance for the linear regression model, with performance quantified as an adjusted *R* ^2^ score; and (*bottom*) the data-derived putative regulatory roles of each histone modification, with positive/negative loadings suggesting activator/repressor roles, respectively. Of particular interest is the latent signature of DNA methylation-associated gene silencing, with GM12878 and K562 exhibiting a higher proportion of near-zero expression genes and strikingly stronger regulatory signal for H3K9me3 (implicated in DNA *de novo* methyltransferase activity)

Using a regression-based gene expression modelling framework [4], we evaluated the accuracy of predicted RPKM-normalised transcript abundance compared to actual RNA-seq data genome-wide for H1-hESC, GM12878 and K562 cell lines. These results are presented in Fig. 2. The performance of these models (adjusted *R*^2^ = 0.43 for H1-hESC and KM562, 0.47 for GM12878) are comparable to those of previous studies [9, 10, 14, 15], supporting our data pre-processing steps and the formulation of our histone score (Equation 1) and regression model (Equation 2).

Figure 2 also presents the distribution of gene expression levels and data-derived regulatory roles of each histone modification genome-wide, with positive/negative loadings suggesting activator/repressor roles, respectively. The data-derived roles reflect the well-established associations between promoter-localised H3K4me3, H3K27me3 and H3K9me3 with respect to genome-wide transcriptional activity. However, it is evident that this approach is unable to identify a consistent role for the H2A.Z histone variant (repressor in dissimilar H1-hESC/K562 and activator in GM12878), which we hypothesise is due to its conditional associations with H3K4me3 and H3K27me3 (promoter bivalency).

Interestingly, the differentiated lymphoblastoid GM12878 and cancerous K562 cell lines exhibit more near-zero expression (silenced) genes than pluripotent H1-hESC, consistent with an increase in DNA methylation-associated gene silencing events during lineage commitment and carcinogenesis. DNA methylation is further implicated by the stronger regulatory signal for H3K9me3 in GM12878 and K562, which is associated with DNA *de novo* methyltransferase activity [16]. It is also interesting to note that principal component 2 (PC2; for which the loadings are presented in Fig. 2) was consistently the only accurate predictor of gene expression (adjusted *R*^2^_PC2_ > 0.4, adjusted *R*^2^_PCx_ < 0.05 ∀ *x* ≠ 2), despite PC1 capturing the most variation in the histone score matrix, **A**, by definition. The orthogonality of PCA thus suggests that PC1 may capture a (linearly uncorrelated) functional signature of histone modification coordination unrelated to transcriptional regulation (e.g. DNA replication [17, 18] or repair [19, 20]).

### MMFS-quantified promoter methylation is anti-correlated with gene expression

**Fig. 3:**
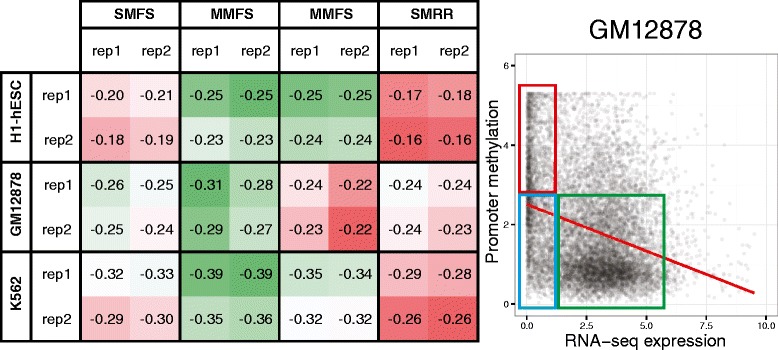
Evaluation of gene-level DNA methylation scores (SMFS, MMFS, MMFR and SMRR). (*Left*) MMFS exhibits the strongest overall anti-correlation with RPKM-normalised transcript abundance (Pearson’s *r* = −0.39), indicating that it is most appropriate for capturing the gene silencing effect of promoter-localised methylation. Model performance is colour-coded by correlation within each cell line, with the best/worst-performing models highlighted in *green*/*red*, respectively. (*Right*) promoter methylation (MMFS) versus transcript abundance genome-wide for GM12878 (regression line shown in *red*), demonstrating two distinct gene clusters: active/unmethylated (*green*) and silent/methylated (*red*). It is also evident that a large number of genes exhibit near-zero expression despite a lack of substantial DNA methylation (*blue*); these genes reduce the predictive power of DNA methylation genome-wide and are likely silenced by other mechanisms (e.g. repressor/silencer transcription factors [22] or H3K27me3-associated Polycomb activity [16])

It is widely accepted that promoter-localised CpG methylation prevents the initiation of eukaryotic gene transcription [21]. By extension, a suitable gene-level DNA methylation score should be anti-correlated with transcript abundance derived from genome-wide RNA-seq data. Figure 3 presents the correlation between transcript abundance and the four DNA methylation scores proposed in this study (sum of methylation fractions by site (SMFS), mean methylation fraction by site (MMFS), mean methylation fraction by region (MMFR) and sum of scaled methylation reads by region (SMRR)) for all replicate combinations. MMFS performed equal-best for H1-hESC (Pearson’s *r* = −0.25) and outright best for GM12878 and K562 (Pearson’s *r* = −0.31 and −0.39, respectively), with all scores exhibiting stronger anti-correlation in the differentiated cell lines than hESC as expected from the previous discussion.

The distribution of promoter methylation (MMFS) versus transcript abundance presented in Fig. 3 demonstrates two distinct clusters, corresponding with active/unmethylated (green) and silenced/methylated genes (red). It is also evident that many genes exhibit near-zero expression despite a lack of substantial DNA methylation (blue), which we attribute to repression driven by other regulatory mechanisms (e.g. repressor/silencer transcription factors [22] or H3K27me3-associated Polycomb activity [16]). This figure illustrates the need to identify and isolate these latent regulatory classes in order to accurately identify the associations between epigenetic regulators and genome-wide mRNA transcript abundance.

### Naïve model integration is unsuitable for DNA methylation data

As demonstrated in Fig. 3, all four gene-level DNA methylation scores are anti-correlated with genome-wide RNA transcript abundance, as expected due to the well-established silencing role of promoter-localised CpG methylation [21]. Intuitively, integrating any of these scores into a gene expression model (particularly MMFS) should yield improved prediction accuracy due to the addition of information regarding methylation-associated silencing.

A naïve approach to integrating DNA methylation into a predictive gene expression model would amount to simply concatenating the vector of methylation scores as a new column of the *n* × *m* histone score matrix, **A**, where *n* is the number of genes and *m* is the number of histone modifications. In this study, **A** contains histone scores for H3K4me3, H3K27me3, H3K9me3 and the H2A.Z histone variant, as described in the ‘Methods’ section. We constructed such models for all combinations of cell line and DNA methylation score and found that the resultant improvement in prediction accuracy was negligible in all cases (|adj. *R*^2^| ≤ 10^−3^).

Despite the anti-correlation shown between each methylation score and RNA transcript abundance, the naïve integration of this information into predictive models trained on histone modification data yields practically zero improvement in prediction accuracy (irrespective of score or cell line). Within the constraints of a linear model, DNA methylation and the four considered histone modifications are statistically redundant with respect to gene expression. This may be partially due to the well-established negative associations between DNA methylation and H3K4me3/H2A.Z [23–25] and positive associations with H3K9ac [16], and we have previously explored the causes of similar redundancy between transcription factor and histone modification data [11]. In the following section, we propose a new framework designed to model the conditional relationships underlying this redundancy, both to provide new insights regarding transcriptional regulation and to allow the information content of DNA methylation data to be effectively leveraged in future integrative studies.

### Modelling transcriptional regulation of methylated and bivalent promoters

To explore the hypothesis that statistical redundancy between histone modification and DNA methylation data is caused by conditional relationships in methylated promoters, the MMFS score was selected to separate genes into two latent regulatory classes (MMFS^+^ versus MMFS^−^) on the basis of a threshold determined through the unsupervised approach described in the methods. Intuitively, this approach should isolate genes subject to H3K9me3/DNA methylation-associated silencing from an otherwise-heterogeneous set.

**Fig. 4:**
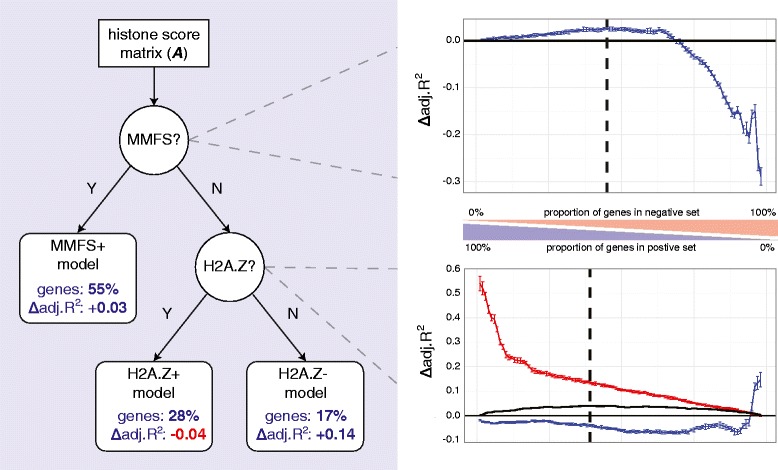
Decision tree of predictive models for the K562 cell line, constructed from the same data as the standard model evaluated earlier (H3K4me3, H3K27me3, H3K9me3, H2A.Z and DNA methylation). This tree uses promoter-localised DNA methylation (MMFS) and the H2A.Z histone variant to classify genes into three latent regulatory classes: MMFS^+^ (high MMFS score), H2A.Z^+^ (low MMFS and high H2A.Z) and H2A.Z^−^ (low MMFS and low H2A.Z). Sub-categorising MMFS^+^ by H2A.Z would be biologically meaningless as the histone variant is mutually exclusive with DNA methylation *in vivo* [25]. Thresholds were learned directly from the data using our unsupervised methodology. Specifically, the *blue*, *red* and *black* lines illustrate Δadj. *R* ^2^ (relative to a standard model constructed from the same data) as a function of threshold values for positive (MMFS^+^), negative (MMFS^−^) and cumulative models, respectively, with the optimal value for both forks indicated by a *black dashed line. Error bars* capture the standard error of the mean (μ ≈ 0) for models constructed from 100 randomly-sampled gene sets of equal size, illustrating the performance variation expected by chance (i.e. fewer genes equals larger variation in model performance, as expected)

Unmethylated genes are still subject to a variety of transcriptional regulatory mechanisms, including H3K4me3-associated euchromatinisation (activation) and H3K27me3-associated facultative heterochromatinisation (repression) [26]. H2A.Z was chosen from the remaining set of epigenetic markers (H3K4me3, H3K27me3, H3K9me3 and H2A.Z) to further separate the set of MMFS^−^ genes into two subclasses (H2A.Z^+^ and H2A.Z^−^) due to our earlier observations and other studies supporting its bivalent regulatory role [27–29]. We believe that histone bivalency confounds the regulatory roles of H3K4me3 and H3K27me3 by maintaining these otherwise-antagonistic markers in metastable equilibrium. The final decision tree structure constructed to test these hypotheses is shown in Fig. 4.

In addition to the decision tree structure, Fig. 4 also demonstrates the following for the K562 cell line: the unsupervised threshold selection process for the proportion of genes attributed to each latent regulatory class; and the respective performance results (Δadj. *R*^2^) relative to a standard predictive model constructed from the same data. The statistics across all ENCODE Tier1 cell lines are presented in Table 1.

**Table 1:** Proportion of genes attributed to each latent regulatory class and respective improvement in prediction accuracy, Δadj *R* ^2^ (relative to a standard model constructed from the same data) for h1-hESC, GM12878 and K562 cell lines

	MMFS^+^		H2A.Z^+^		H2A.Z^−^	
	Genes (%)	Δadj. *R* ^2^	Genes (%)	Δadj. *R* ^2^	Genes (%)	Δadj. *R* ^2^
H1-hESC	46	+0.03	25	−0.01	28	+0.05
GM12878	40	+0.06	29	−0.13	30	+0.16
K562	55	+0.03	28	−0.04	17	+0.14

By separating genes into regulatory classes based on the latent signature of methylated and bivalent promoters, it is evident from Table 1 that the inferred relationships between epigenetic and expression data are strengthened for the majority of genes; e.g. 55 and 17 % of K562 genes are classified as MMFS^+^ and H2A.Z^−^, and our ability to predict the expression of these genes improves substantially (Δadj. *R*^2^ = 0.03 and 0.14, respectively).

We note that H2A.Z^+^ genes are actually reduced in prediction accuracy, particularly in GM12878. We speculate that this is due to further latent subclasses of H2A.Z-associated regulation. H2A.Z flanks the TSS during transcriptional activation but is evicted during transcript elongation [30, 31], suggesting that a temporal model may be necessary to fully resolve its complex regulatory role. It is also likely that the collection and integration of RNA polymerase II CTD-S2 phosphorylation data (an indicator of paused elongation at H2A.Z-flanked CpG islands [32]) would improve our ability to precisely model this process. Irrespectively, this study has provided an unsupervised framework for identifying these difficult-to-model genes and demonstrated that the occurrence and regulatory signature of promoter bivalency changes significantly following lineage commitment and carcinogenesis. A list of the regulatory classes assigned to each gene across all ENCODE Tier1 cell lines is provided in Additional file 1, demonstrating substantial poised ↔ active plasticity compared to greater stability in DNA methylation-associated silencing. These findings are of particular importance in the context of recent studies linking H2A.Z over-expression to the progression of various cancers [33–35].

Importantly, previous downstream analyses [9–12] can be applied to any latent regulatory class-specific gene set in isolation (MMFS^+^, H2A.Z^+^, H2A.Z^−^), allowing researchers to investigate regulatory activity specific to a particular chromatin context. Although this study has utilised prior knowledge to structure a decision tree specific to our regulatory context of interest (methylated and bivalent promoters), the approach can be extended to an arbitrary set of epigenetic markers by systematically evaluating all possible tree structures.

## Conclusions

Predictive gene expression modelling is an essential tool in computational biology, due to both the critical importance of understanding transcriptional regulation and the current inability to source sufficient proteomics to resolve data on a gene-by-gene basis. However, we believe that previous predictive models designed to explore regulatory activity have been confounded by conditional associations between epigenetic regulators and gene expression within a dynamic chromatin context. This study extends our previous investigation of statistical redundancy between transcription factors and histone modifications [11], which concluded that the principled integration of additional epigenetic variables (e.g. DNA methylation and H2A.Z) would be necessary to effectively model these processes.

Our results demonstrate that the naïve integration of DNA methylation data into a standard predictive model is unable to improve prediction accuracy despite strong anti-correlation between gene expression and our proposed gene-level methylation scores, strongly suggesting that more complex regulatory logic is involved. Instead, we present a new modelling framework that substantially improves genome-wide predictions of mRNA transcript abundance by using DNA methylation data to identify and separate genes into latent regulatory classes. These improvements were demonstrated across all ENCODE Tier1 cell lines [8], using a minimal set of epigenetic markers (H2A.Z, H3K4me3, H3K9me3 and H3K27me3) chosen for their indirect and context-sensitive regulatory roles in a promoter-localised context.

The improved performance across multiple dissimilar cell lines supports our hypothesis that statistical redundancy in epigenetic data is caused by conditional associations between regulators and gene expression. In addition to leveraging DNA methylation data to identify the latent signature of epigenetically silenced genes, we identified the conditional, bivalent associations of H3K4me3 and H3K27me3 with the H2A.Z histone variant. Although predictive models at bivalent promoters remain less accurate than for non-bivalent genes, this study has provided a framework for identifying these genes and demonstrated that the occurrence and regulatory signature of promoter bivalency changes substantially across the lineage commitment spectra (from H1-hESC to GM12878) and carcinogenesis (K562). These findings are of particular importance in the context of recent studies linking H2A.Z over-expression to the progression of various cancers [33–35].

It is well-established in previous literature that the utility of gene expression models extends beyond the ability to predict the expression levels of individual genes. The interpretability of these models across all biological problems is, however, limited by prediction accuracy at this level. As well as allowing us to investigate gene expression at methylated and bivalent promoters, we anticipate that this interpretation of conditional regulatory activity in predictive models will improve the explorative potential of future *in silico* studies in two fundamental ways. Firstly, the accuracy of gene expression models is substantially improved by unsupervised separation of genes into latent regulatory classes. Secondly, researchers have the option of specifying classification variables from prior knowledge (the approach demonstrated in this study) to investigate gene regulatory logic specific to a particular chromatin context (e.g. the role of pioneer transcription factors in euchromatin versus heterochromatin). Alternatively, all combinations of classification variables can be exhaustively evaluated to provide a fully generalisable and unsupervised analysis.

## Methods

### Cell line data

ENCODE Tier1 cell lines (H1-hESC, GM12878 and K562) were selected to explore methylated and bivalent promoters, as functional patterns of DNA methylation vary substantially during lineage commitment and carcinogenesis. All cell line gene expression (RNA-seq), histone modification (ChIP-seq) and DNA methylation (methyl RRBS) data were downloaded from ENCODE [8]. Specific GEO accession numbers for each dataset are provided in Table 2. The TSS for each gene was taken from the gene annotation dataset for the human genome (hg19/GRCh37). Multiple transcripts or isoforms were removed by considering only the most 5′-located TSS for each unique Ensembl gene identifier, resulting in a set of 11,806 genes with unambiguous mappings. RNA-seq data was re-mapped to hg19 using Subread [36] and RPKM-normalised using edgeR [37, 38].

**Table 2:** All ENCODE Tier1 cell line data used in this study [8]

Data type	Data source
RNA-seq	GSM958730 (GM12878, 2 replicates)
	GSM958737 (H1-hESC, 2 replicates)
	GSM958731 (K562, 2 replicates)
TSS	Ensembl hg19/GRCh37 [44]
Methyl RRBS (GM12878)	GSM683906 (replicate 1)
	GSM683927 (replicate 2)
ChIP-seq (GM12878)	GSM733767 (H2A.Z)
	GSM733758 (H3K27me3)
	GSM733708 (H3K4me3)
	GSM733664 (H3K9me3)
Methyl RRBS (H1-hESC)	GSM683770 (replicate 1)
	GSM683879 (replicate 2)
ChIP-seq (H1-hESC)	GSM1003579 (H2A.Z)
	GSM733748 (H3K27me3)
	GSM733657 (H3K4me3)
	GSM1003585 (H3K9me3)
Methyl RRBS (K562)	GSM683856 (replicate 1)
	GSM683780 (replicate 2)
ChIP-seq (K562)	GSM733786 (H2A.Z)
	GSM733658 (H3K27me3)
	GSM733680 (H3K4me3)
	GSM733776 (H3K9me3)

### Gene-specific histone modification scores

The association strength between a gene, *i*, and histone modification, *j*, is calculated using the constrained sum-of-tags histone score [4]:

1 $$ {a}_{ij}={\displaystyle \sum_k}{g}_k, $$

where *g*_*k*_ is the number of ChIP-seq reads (or normalised equivalent) for *j* mapped to position *k* relative to the TSS of *i*. As ChIP-seq involves sequencing of DNA corresponding with the end of each nucleosome, the position for each read was shifted by ±73 bp (for ± strand, respectively) to centre on the modified nucleosome [15]. Integrating over a region 2000 bp either side of the TSS (approximating the average width of histone modification ChIP-seq binding regions) is standard for this approach [9, 10, 14] and applied throughout this study.

### Gene-specific DNA methylation scores

Compared to CpG-level methylation scores, gene/region-level DNA methylation scores are not well-established in previous literature. We explore four possible promoter-localised scores in the context of predictive gene expression modelling, considering a window 2000 bp either side of the respective gene’s TSS:

- Sum of methylation fractions by site (SMFS): Sum of the CpG-level methylation scores within a region, similar to the constrained sum-of-tags score previously applied to the analysis of ChIP-seq data [4]
- Mean methylation fraction by site (MMFS): Equivalent to the SMFS score divided by the number of assayed CpGs within the region, similar to the mean methylation level described by Shultz et al. [39]
- Mean methylation fraction by region (MMFR): proportion of raw reads that were found to be methylated, similar to the weighted methylation level described by Schultz et al. [39]
- Sum of scaled methylation reads by region (SMRR): Equivalent to the MMFS score except each read is multiplied by − exp(*d*/*d*0), where *d* is the distance (bp) from the TSS and *d*0 = 5000, similar to the exponentially decaying affinity score previously applied to the analysis of ChIP-seq data [4]

### Regression-based predictive modelling of gene expression

In this study, we model the RPKM-normalised transcript abundance, *y*_*i*_, of each gene, *i*, as a general linear function of its association, *a*_*ij*_, with each histone modification, *j*:

2 $$ { \sinh}^{-1}\left({y}_i\right) = \mu +{\displaystyle \sum_j}{\beta}_j{a}_{ij} + {\varepsilon}_i, $$

where *β*_*j*_ captures the influence of histone modification *j* on gene expression, *μ* is the basal expression level, and *ε*_*i*_ is the gene-specific error term. The inverse hyperbolic sine (arsinh) transformation, ${ \sinh}^{-1}(x)= \log \left(x+\sqrt{1+{x}^2}\right),$ is approximately equal to log (2*x*) for *x* ≫ 0, allowing it to be regarded as practically equivalent to the log-transformation applied in previous gene expression modelling studies [4]. Unlike log (*x*), sinh^−1^(*x*) is defined for *x* = 0, removing the need to meta-optimise small constant to add to *x* (leading to spurious inflation of reported prediction accuracy) and making it better-suited to integrating ChIP-seq and RPKM-normalised RNA-seq data.

### Evaluation of prediction accuracy

Prediction accuracy is assessed for each regression model using an adjusted *R*^2^ score, which in comparison to the standard *R*^2^ approach prevents spurious inflation of the statistic due to the introduction of additional explanatory variables [40]. Separate RNA-seq replicates and cross-validation were used for model training and evaluation to prevent over-fitting to experimental noise where appropriate.

### Derivation of putative regulatory roles

Putative regulatory roles are inferred for each histone modification using principal component analysis (PCA). Specifically, the histone score matrix, **A** (see Equation 1), for a gene set of interest is arsinh-transformed (see Equation 2) and reformulated using the following singular value decomposition [41]:

3 $$ { \sinh}^{-1}\left(\mathbf{A}\right)=\mathbf{U}\boldsymbol{\Sigma } {\mathbf{V}}^{\mathrm{T}}, $$

where **U** is the matrix of component scores, **Σ** is the diagonal matrix of the singular values of **A**, and **V** is the matrix of loadings (weights by which the histone scores are multiplied to derive their respective component scores). In the context of modelling gene expression, the columns of the matrix **UΣ** are the principal components (PCs), and the rows correspond with eigengenes [42]. The data-derived regulatory role of each histone modification is simply its contribution (loading) toward the individual PC most predictive of gene expression [4].

### Modelling conditional regulatory interactions with decision trees

**Fig. 5:**
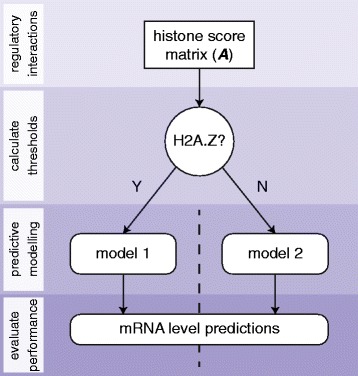
Illustration of our predictive modelling approach where the H2A.Z histone variant has been used to separate genes into two classes. Categorising genes by the presence of promoter-localised H2A.Z removes context sensitivity in the regulatory role of H3K4me3; H3K4me3 in the presence of H2A.Z is often a hallmark of low expression (i.e. bivalent genes), whereas H3K4me3 is otherwise associated with active transcription. These conditional interactions are poorly handled by previous regression models

We provide a framework for improved modelling of conditional and synergistic interactions from matched transcriptomic and epigenomic data. As an illustrative example, gene-level H2A.Z scores (an indicator of histone bivalency) could be used to separate genes into two subsets: those associated with H2A.Z (bivalent) and those that are not. Separate predictive models can then be constructed and evaluated for both subsets from the remaining regulatory elements (i.e. no longer using H2A.Z as a predictor), as illustrated in Fig. 5; statistical artefacts introduced by the reduced degrees-of-freedom are corrected by the adjusted *R*^*2*^ evaluation metric [40].

This model is implemented in a binary decision tree, where each node represents a linear regression model over a subset of genes and each non-leaf node represents a categorisation step (e.g. associated with H2A.Z (bivalent) or not). The genes associated with each leaf node partition the full set of genes from the histone score matrix, **A**, and the respective models are presumed to capture homogeneous regulatory logic. Regression models associated with non-leaf nodes are only used for the assignment of categorisation thresholds.

We use an unsupervised method to define the threshold, γ, above which a gene-level histone score is accepted to represent actual regulatory activity. More formally, given an *n* × *m* matrix **A** of histone scores (*n* genes by *m* histone modifications), histone modification vector *A*_*x*_^*T*^ (*x* ∈ [1, *m*]) is selected to partition **A** into two regulatory classes, **A**_*k* × *m* − 1_^left^ and **A**_*n* − *k* × *m* − 1_^right^, where *k* is the number of genes for which *A*_*x*_^*T*^ ≤ *γ*. In a tree-based representation where **A**_*k* × *m* − 1_^left^ and **A**_*n* − *k* × *m* − 1_^right^ are the left and right children of **A**, respectively, there are two scenarios for the unsupervised assignment of the threshold *γ*:

- If exactly one child of the H2A.Z node is a leaf, the threshold is optimised to maximise the prediction accuracy of the respective model. This model is assumed to capture genes subject to homogeneous regulation.
- Otherwise, the threshold is optimised to maximise the prediction accuracy of both left and right child models (i.e. the adjusted *R*^2^ calculated over the concatenated predictions for both regulatory classes).

This approach can be repeated recursively for a binary decision tree of arbitrary height (constrained by the number of epigenetic variables, *m*) and balance. At each step, the selection of which variable is used to partition **A** into separate models can be either manually selected to explore specific regulatory contexts (the approach demonstrated in this study) or automated to exhaustively evaluate all possible tree structures (computationally tractable for a practical number of matched epigenetic datasets). In the latter scenario, the greedy nature of the threshold selection procedure still does not guarantee a globally optimal set of values; improved prediction accuracy could be obtained by multivariate optimisation across the full set of non-leaf thresholds for an arbitrarily large tree, although we argue that this approach would a) lose the biological meaning (regulated-or-not) underlying the methodology presented, and b) be poorly suited to the recursive implementation preferable for tree-based algorithms.

### Implementation

All scripts used in this study are implemented using open-source software and made available as a pre-configured bootable virtual environment [43]. This environment was created using a minimal installation of Lubuntu 13.10; a lightweight Linux distribution which supports all the tools required. R version 3.0.1 was installed, along with the core set of packages and utilities required to explore the presented results. This environment, along with all data and scripts, are available online at http://sourceforge.net/projects/budden2015treeome/.

## Additional file

Additional file 1:
**Regulatory classes. Regulatory class assigned to each gene across all ENCODE Tier1 cell lines.**

## Abbreviations

ChIP: Chromatin immunoprecipitation
CpG: Cytosine-guanine dinucleotide
ENCODE: Encyclopedia of DNA elements
ESC: Embryonic stem cell
GM12878: Human lymphoblastoid cell line
hESC: Human ESC
K562: Human leukaemia cell line
MMFR: Mean methylation by region
MMFS: Mean methylation fraction by site
PCA: Principal component analysis
PWM: Position weight matrix
RPKM: Reads per kilobase per million
SMRR: Sum of scaled methylation reads by region
SMFS: Sum of methylation fractions by site
TSS: Transcription start site
